# *Ascaris suum* infection in juvenile pigs elicits a local Th2 response in a setting of ongoing Th1 expansion

**DOI:** 10.3389/fimmu.2024.1396446

**Published:** 2024-05-10

**Authors:** Larissa Oser, Ankur Midha, Josephine Schlosser-Brandenburg, Sebastian Rausch, Robert M. Mugo, Arkadi Kundik, Luis E. Elizalde-Velázquez, Joshua Adjah, Zaneta D. Musimbi, Robert Klopfleisch, Christina S. Helm, Georg von Samson-Himmelstjerna, Susanne Hartmann, Friederike Ebner

**Affiliations:** ^1^ Centre for Infection Medicine, Department of Veterinary Medicine, Institute of Immunology, Freie Universität Berlin, Berlin, Germany; ^2^ Department of Veterinary Medicine, Institute of Veterinary Pathology, Freie Universität Berlin, Berlin, Germany; ^3^ Department of Veterinary Medicine, Institute for Parasitology and Tropical Veterinary Medicine, Freie Universität Berlin, Berlin, Germany; ^4^ Infection Pathogenesis, School of Life Sciences, Technical University of Munich, Freising, Germany

**Keywords:** pig, soil-transmitted helminth, Ascaris suum, antigen-specific, adaptive immunity, infection, protection, Th1/Th2

## Abstract

*Ascaris* spp. undergo extensive migration within the body before establishing patent infections in the small intestinal tract of humans and pigs. However, whether larval migration is critical for inducing efficient type 2 responses remains poorly understood. Therefore, we investigated systemic versus local adaptive immune responses along the hepato-tracheal migration of *Ascaris suum* during primary, single infections in conventionally raised pigs. Neither the initial invasion of gut tissue nor migration through the liver resulted in discernable Th2 cell responses. In contrast, lung-stage larvae elicited a Th2-biased pulmonary response, which declined after the larvae had left the lungs. In the small intestine, we observed an accumulation of Th2 cells upon the arrival of fourth-stage larvae (L4) to the small intestinal lumen. In parallel, we noticed robust and increasing Th1 responses in circulation, migration-affected organs, and draining lymph nodes. Phenotypic analysis of CD4+ T cells specifically recognizing *A. suum* antigens in the circulation and lung tissue of infected pigs confirmed that the majority of *Ascaris*-specific T cells produced IL-4 (Th2) and, to a much lesser extent, IL-4/IFN-g (Th2/1 hybrids) or IFN-g alone (Th1). These data demonstrate that lung-stage but not the early liver-stage larvae lead to a locally restricted Th2 response. Significant Th2 cell accumulation in the small intestine occurs only when L4 complete the body migration. In addition, Th2 immunity seems to be hampered by the concurrent, nonspecific Th1 bias in growing pigs. Together, the late onset of Th2 immunity at the site of infection and the Th1-biased systemic immunity likely enable the establishment of intestinal infections by sufficiently large L4 stages and pre-adult worms, some of which resist expulsion mechanisms.

## 1 Introduction

The large roundworm *Ascaris suum (A. suum)* is one of the most relevant and prevalent gastrointestinal, soil-transmitted helminths in swine worldwide (1–5). Due to its zoonotic potential, its eradication is a major One Health concern (6). Moreover, *Ascaris lumbricoides* (*A. lumbricoides*) the human roundworm can also develop patent infections in swine and is genetically almost identical with *A. suum* (7). The transmission of *Ascaris* spp. is mediated through the fecal-oral route following the development of infective third-stage larvae (L3) in the egg. Once infective eggs are ingested, larvae hatch in the intestine and undergo a body migration through the liver and lungs before returning to the intestine, where they mature and develop into fertile adults (8). Infection can lead to severe damage of liver tissue, acute pneumonia following larval lung passage (9), and heavy adult worm infestations can obstruct the intestine (10). The more frequently observed low parasite burden leads to chronic infections, and based on the extraordinary fecundity of female worms to the constant shedding of large numbers of eggs, mostly in the absence of clinical symptoms (11). In the infected host liver tissue granulomas, referred to as milk spots, form as a response to migrating larvae and lead to liver condemnation upon slaughter (11, 12). In addition, ascariasis results in a reduced feed conversion and growth rate in pigs, next to a potential impact on the outcome of secondary infections, and vaccination responses that together result in relevant economic losses in the porcine industry (13–16). Current strategies targeting the control of *A. suum* rely on the use of anthelmintics and hygiene measures (17). Despite being highly efficient, the long-term benefits of using anthelmintics are disappointing as reinfections occur regularly (18). Thus, there is a strong demand for vaccinations to prevent the development of patent infection and enable long-term control of the disease. However, the immune mechanisms, in particular the adaptive immune responses generated during larval migration, are not yet well understood.

In general, the immune control of intestinal helminthiasis depends on the balance between Th2 and Th1 cells (19–21). Data from patients infected with *A. lumbricoides*, showed that current high-density infection (high egg counts in feces) was associated with elevated production of the type 2 cytokines interleukin (IL)-5 and IL-13, while IFN-γ levels were similar as in uninfected patients (7, 22). The type 2 profile was deemed important for the control of the infection, as patients displaying robust type 2 responses were less likely to be reinfected upon drug cure in longitudinal studies (23). Furthermore, murine models of *Heligmosomoides polygyrus* (*H. polygyrus*) and *Trichuris muris* (*T. muris)* clearly showed that resistance rises along the genetically determined competence for Th2 immune response and that the extent of Th2 responses determines the kinetic of worm expulsion (24, 25). Conversely, enforced type 1 signaling via IL-12/IFN-γ resulted in the delayed control of infections (26). Similarly, our group has shown that the age-dependent increase in IFN-γ competence interferes with the formation of an effective type 2 response in *H. polygyrus* infected mice, resulting in impaired parasite control at an advanced age (27). Moreover, laboratory mice that were transferred from pathogen-free to outdoor environments lost the ability to develop sterile immunity against the nematode *T. muris*, which was associated with an immune response shifted towards Th1 and thus increased susceptibility (28).

Meanwhile, data on the cellular immunity against *A. suum* in domestic swine are sparse. Ascariasis in swine is accompanied by immune cell infiltration in liver and lung tissue as well as blood eosinophilia (29, 30). Increased IL-4 plasma levels and *Ascaris*-specific IL-4 production in the systemic circulation were reported for primary and trickle-infected pigs (31, 32). Transcriptomic studies have indicated an increase in Th2-associated genes (*IL-4, IL-5, IL-13*) in the tissues affected by migration, namely the liver, lung, and jejunum (31, 33).

Despite type-2 associated gene and cytokine signatures, constant reinfections are observed in many natural hosts of soil-transmitted helminths and are well documented for both *A. lumbricoides* and *A. suum* in humans and pigs, respectively (34, 35). This is surprising, considering the multiple barriers confronted by the larval stages while undergoing body migration through several organ systems. While body migration comes with many disadvantages such as high energy expenditure, potential attrition, and the constant need to adapt to various physiological environments, it also favors parasite survival (36). Tissue migration has been shown to lead to increased parasite size and speed of maturation (37). Moreover, it has been shown that type 2 responses are more efficient against adult, mucosal-dwelling life stages than migratory larval stages, and thus tissue-migration allows the parasite to avoid specific host immune-defense mechanisms (36, 37). However, whether *A. suum* larval migration might serve as an immune evasion from anti-parasite responses and which adaptive cellular immune responses are induced during migration is poorly understood. Therefore, the current study assessed the phenotypical composition of the CD4^+^ T helper cell pool following primary, single *A. suum* infection in juvenile pigs.

Our data from experimental infections revealed that Th2 responses are largely confined to mucosal tissues, but not prominently seen in the liver or systemic circulation. This response partially resolved in the lungs after larval passage, but only followed in the small intestine after the establishment of luminal enteric infection by the premature adult stage. Concurrently, we found a rise of Th1 cells in various organs as well as in the systemic circulation during primary *A. suum* infection. In contrast, *Ascaris*-specific T helper cells identified via CD40L expression predominantly displayed an IL-4+ phenotype in the lung tissue as well as in the circulation.

Together, our data indicate that the swift migration of the hatched larvae across the intestinal epithelial barrier does not result in a discernable accumulation of Th2 cells in the intestine, thus we speculate that the final target organ is left unprepared for the returning larvae. This contrasts with the lung, where larval migration leads to a locally restricted Th2 response, followed by a similar, but delayed type profile expressed in the small intestine after the larvae returned to the gut. Despite the formation of an *Ascaris*-specific Th2 immune response, ubiquitous, parasite non-specific type 1 immune signatures might hinder the establishment of a robust and stable type 2 immune response against *A. suum* across the affected organs, thus contributing to constant reinfection observed in natural hosts.

## 2 Materials and methods

### 2.1 Ethics statement

All animal experiments were carried out at Freie Universität Berlin under the principles outlined in the European Convention for the Protection of Vertebrate Animals used for Experimental and Other Scientific Purposes and the German Animal Welfare Law. Ethical approval was provided by the State Office of Health and Social Affairs (Landesamt für Gesundheit und Soziales) in Berlin, Germany for obtaining blood and tissues from healthy uninfected pigs, for generating parasite antigen material and for experimental infection of pigs (G0278/18, H0005/18, T0002/18).

### 2.2 Generation of infective *A. suum* eggs and parasitic antigens

Infective *A. suum* eggs were generated as previously described (38). In brief, after culturing female adult worms obtained from a local abattoir overnight in worm culture medium (Hank´s Balanced Salt Solution (HBSS) supplemented with 200 U/ml Penicillin, 200 µg/ml Streptomycin, 50 µg/ml Gentamycin, and 2.5 µg/ml Amphotericin B, all PAN Biotech) at 37°C and 5% CO_2_, eggs were collected from the culture supernatant. Eggs were washed several times in HBSS, and placed in 0.1% formalin-containing distilled water for embryonation (6-8 weeks, 33°C, protected from light). Embryonation rates were assessed microscopically and eggs with approximately 94% embryonation rates were used for infection experiments.

Adult *A. suum* lysate for antigen-specific restimulation was prepared by dissecting male and female adult *A. suum* worms that were collected from a local abattoir. Ice-cold PBS (PAN Biotech) was added to the minced worms before further homogenization. Subsequently, samples were centrifuged and passed through a 0.45µm filter. Before third stage (L3) *A. suum* lysate preparation, larvae were hatched as described in Kundik et al. (39). Pellets of hatched larvae were then resuspended in PBS, shredded, and centrifuged. The L3 homogenate was then frozen at -80°C before sonification, centrifugation, and filtration through a 0.22µm filter. The protein content of both lysates was measured via Bicinchoninic acid (BCA) assay as previously described (40).

### 2.3 Animals and experimental infections

German landrace hybrid pigs of both sexes were obtained from conventional breeding facilities (Brandenburg and Saxony district, Germany) at 5-7 weeks of age. Water was given ad libitum and food was provided twice a day according to the pig’s body weight which was assessed weekly. All pigs were allowed to acclimatize for one week before experimental infection.

Pigs were housed in indoor stables with controlled light, temperature, and ventilation systems.

Study 1: To evaluate the systemic Th2 and Th1 response kinetics in blood, 5 pigs were infected orally with a dose of 4000 A*. suum* eggs in bread rolls. Blood was collected weekly from the external jugular vein up to 7 weeks post-infection (wpi).
Study 2: To evaluate Th2 and Th1 dynamics in migration-affected tissues, 17 pigs were infected orally with a dose of 4000 A*. suum* eggs in waffles. Animals were dissected at 2 wpi (n=5), 4 wpi (n=6), and 5 wpi (n=6), while one group of 5 animals served as naïve control. Baseline blood samples were taken from all pigs before infection (0 wpi).
Study 3: To evaluate *Ascaris*-specific T cells in blood and lung tissue, 8 pigs were infected orally with a dose of 4000 A*. suum* eggs by oral gavage. Pigs were dissected at 7 days post-infection (dpi; n=4) and 12 dpi (n=4).

### 2.4 Parasitological examination

The presence and quantity of *A. suum* eggs shed in feces were analyzed as previously described using the Mini-FLOTAC method (Unit of Parasitology and Parasitic Diseases at the University of Naples Federico II) (41). For screening, 5.0-5.5 g of fecal material was pooled from all pigs of a group, and for determining fecal egg counts (FEC) per animal, individual samples were taken. Fecal samples were mixed with 45ml saturated saline solution and homogenized. Samples were passed through a 500µm sieve and filled into Mini-FLOTAC chambers. Two chambers were analyzed for each sample according to the manufacturer’s protocol.

Larval stages (4 wpi), pre-mature worms (5 wpi), and adult worms (8 wpi) were collected after macroscopic inspection and counted from the entire intestine of each pig, then measured for length. At 2 wpi, larval stages were too small for macroscopic examination.

### 2.5 Necropsy and tissue sampling

Following sedation with ketamine hydrochloride (20mg/kg BW; Ursotamin; Serumwerk Bernburg AF), azaperone (2mg/kg BW; Stresnil; JanssenCilag GmbH) and xylazine (36mg/kg BW; Xylavet; CP-Pharma Handels GmbH), blood was sampled by heart puncture. Animals were then euthanized by intracardial injection of tetracaine hydrochloride, mebezoniom iodide, and embutramide (10mg/kg BW; T61, Intervet, Germany).

Broncho-alveolar lavage (BAL) cells were obtained after clamping the trachea, removing the lung, and flushing the right lung lobe with 200ml of PBS supplemented with 2mM EDTA. Lavage fluid was filtered through a 70µm cell strainer and stored on ice for further processing. Tissue samples were processed as previously described (42). Briefly, two separate samples were collected from the spleen and liver and transferred to RPMI wash solution (1% FCS, 100 U/ml penicillin, and 100 µg/ml streptomycin) and stored on ice. From the lung, three tissue samples were obtained from the caudal left, cranial right, and right accessory lung lobe and stored in wash RPMI at 4°C. Tissue samples of the jejunum were collected near the detected worms (if no worms were present, samples were collected from the proximal-intermediate jejunum) and stored in wash RPMI at 4°C. Lymph nodes were sampled from the lung (cranial, right, middle, and left tracheobronchial), and the jejunum (7-10 mesenteric lymph nodes (mLN) draining the sites where worms were found) and pooled and stored in wash RPMI on ice.

### 2.6 Differential cell counts of blood and BAL

Blood smears were Romanowsky stained (DiffQuick, Labor + Technik, Eberhard Lehmann GmbH, Germany), and 200 cells were counted and classified to determine differential white blood cell (WBC) counts. Percentages of lymphocytes, neutrophils, eosinophils, basophils, and monocytes were calculated. BAL fluid cells were transferred to microscope slides using cytospin (1000 x g, 10 min, RT) and fixed using ROTIFix^®^ spray (Carl Roth) before Romanowsky staining and classification.

### 2.7 Leukocyte isolation procedures from blood, lymphatic, and organ tissues

Mononuclear cells from porcine peripheral blood collected in Lithium-Heparin coated tubes (S-Monovette, Sarstedt) were isolated through density centrifugation of diluted (1:1 in 0.9% saline solution) blood using Pancoll solution (density 1.077g/ml, PAN-Biotech). Cells obtained from the buffy coat layer were washed in 0.9% NaCl. The remaining erythrocytes were lysed using erythrocyte lysis solution (0.01M KHCO_3_, 0.155M NH_4_Cl, 0.1mM EDTA, pH 7.5) for 5 min at RT and lysis was stopped with complete IMD medium (cIMDM, 100 U/mL penicillin, 100 µg/mL streptomycin and 10% fetal calf serum (FCS); all PAN-Biotech). Lymphocytes were pelleted, washed, and resuspended in cIMDM.

Splenic leukocytes were isolated by mechanical disruption of spleen tissue and passing the cell suspension through a 70µm cell strainer. Splenocytes were pelleted and erythrocytes were removed by lysis as described above. Lymphocytes were pelleted, washed, and resuspended in cIMDM.

For lymphocyte isolation from lymph nodes, excessive fat and connective tissue were removed and pooled lymph nodes were cut into small pieces and forced through a 70µm cell strainer by using the plunger from a 5ml syringe. Lymphocytes were pelleted, washed, and resuspended in cIMDM.

For the isolation of liver lymphocytes, several 1 x 1 cm pieces were pooled from two different collection regions for each pig. Tissues were minced and predigested using pre-warmed complete Hank´s balanced salt solution (cHBSS, 2% FCS, 0.6% BSA; all PAN-Biotech) supplemented with 125 U/mL Collagenase VIII (Sigma-Aldrich), 125 U/mL Collagenase D (Sigma-Aldrich) and 0.180 mg/mL DNase I (Sigma-Aldrich) for 20 minutes at 37°C. The cell suspension was filtered through a 190µm sieve and digestion was continued by adding complete HBSS containing 0.180 mg/mL DNase I, 0.125 mg/mL Liberase TM (Sigma-Aldrich), and 0.125 mg/mL Liberase DH (Sigma-Aldrich) for 30 minutes at 37°C. Ice-cold HBSS was used to stop digestion. The suspension was filtered through a 70µm cell strainer. The remaining erythrocytes were lysed and the cells were washed. Gradient centrifugation (453 x g, 20 min, 4°C) was performed by resuspending the liver cells in 40% Percoll solution (GE Healthcare) and transferring them to 70% Percoll. Lymphocytes were collected from the interphase, pelleted, washed, and resuspended in cIMDM.

Lung lymphocyte isolation was performed as previously described (42). In brief, tissue samples from three different lung regions were pooled together and cut into small pieces. Tissue samples were washed and enzymatically digested using 0.125 U/mL Collagenase D, 0.180 mg/mL DNaseI, and 0.125 mg/mL Liberase DH and TM each. The remaining erythrocytes were lysed and cells were washed. Subsequently, gradient centrifugation was performed (40%/70% Percoll). Lymphocytes were collected from the interphase, pelleted, washed, and resuspended in cIMDM.

For isolation of small intestinal lamina propria lymphocytes (siLPL), an approximately 10 x 10 cm tissue sample from the jejunum was cut into small pieces and washed with HBSS (w/o Ca^2+^ and Mg^2+^; Pan-Biotech), containing 10mM HEPES, 16.6mM NaHCO_3_, and 2% FCS (all Carl Roth GmbH + Co.KG). Samples were vortexed and washed with HBSS/EDTA (HBSS containing 10% FCS, 15mM HEPES, and 5mM EDTA, Carl Roth GmbH & Co.KG). EDTA-containing buffers were replaced with LPL-media (RPMI 1640 with 15mM HEPES, 5% FCS, 0.2% gentamycin (Pan Biotech) for 5 min at RT. Tissue samples were cut into fine pieces and digested in LPL-media using 5mg/mL Liberase TM, 5mg/mL Liberase DH, and 4mg/mL DNaseI at 37°C under gentle agitation (2 x 20min). Cells were mechanically disrupted by forcing tissues through a 190µm mesh. Subsequently, gradient centrifugation was performed (40%/70% Percoll). Lymphocytes were collected from the interphase, pelleted, washed, and resuspended in cIMDM.

### 2.8 Flow cytometric immunophenotyping

Freshly isolated lymphocytes were seeded at 3 million cells per well in a 96-well plate and either allowed to rest overnight at 37°C for cytokine staining or directly processed for transcription factor analysis. Cell surface markers were stained using directly labeled pig-specific antibodies, or by applying primary antibodies followed by a secondary isotype-specific antibody or streptavidin. The fixable viability dye eFluor 506 (Thermo Fisher) was used to identify dead cells. Intranuclear and intracellular targets were stained after fixation and permeabilization of cells with the FoxP3/Transcription Factor staining buffer set (Thermo Fisher) or Cytofix/Cytoperm (BD Biosciences), respectively. Staining procedures were performed according to current standards (43). **Table 1** lists all the porcine-specific, and cross-reactive or secondary antibodies used in the study.

**Table 1:** Antibodies used for *ex vivo* flow cytometric phenotyping.

Antigen	Clone	Isotype	Fluorochrome	Labelling strategy	Company
gdTCR1	PGBL22A	mouse IgG1	unlabeled	indirect^a^	Kingfisher Biotech
CD3e	BB23-8E6-8C8	mouse IgG2a or mouse IgG2b	PerCp-Cy5.5 or Biotin	direct indirect^b^	BD Biosciences Southern Biotech
CD4a	74-12-4	mouse IgG2b	FITC or PE-Cy7 or PerCp-Cy5.5	direct	Southern Biotech or BD Biosciences
CD8a	76-2-11	mouse IgG2a	unlabeled or PE	indirect^c^ or direct	ThermoFisher or BD Biosciences
CD14	Tük4	mouse IgG2a	VioGreen	direct	Milteny Biotec
CD27	B30C7	mouse IgG1	PE	direct	Bio-Rad
CD40L	5C8	mouse IgG2a	PE	direct	Milteny Biotec
IFN-γ	P2G10	mouse IgG1	PerCp-Cy5.5 or A647	direct	BD Biosciences
IL-4	MP4-25D2	rat IgG1	PE-Cy7 or FITC	direct	BioLegend
FoxP3	FJK-16S	rat IgG2a	eFluor 450	direct	ThermoFisher
GATA3	TWAJ	rat IgG2b	eFluor 660	direct	ThermoFisher
Ki-67	SolA15	rat IgG2a	Pe-Cy7	direct	ThermoFisher
Tbet	4B10	mouse IgG1	Brilliant Violet 605	direct	BioLegend
Fixable viability dye (DCE)			eFluor 506	direct	ThermoFisher

a anti-mouse IgG1 – RMG1-1, APC-Cy7, BioLegend.

b Streptavidin – Alexa 700, ThermoFisher.

c rat anti-mouse IgG2a – R19-15, Brilliant Violet 605, BD Biosciences.

For analyzing cytokine expression, lymphocytes were left untreated (w/o) or stimulated with Phorbol myristate acetate (PMA; 50 ng/ml, Sigma-Aldrich) plus ionomycin (500 ng/ml, Sigma-Aldrich) for 3.5h in presence of Brefeldin A (1 µg/ml, eBioscience) during the last 3h of restimulation. Cells were acquired on a BD FACS Aria III (BD Biosciences) using the FACSDiva software (BD Biosciences) and evaluated using FlowJo v10 software (Tree Star).

### 2.9 Antigen-specific T cell enrichment for *Ascaris*-specific CD4+ T cells

Magnetic enrichment and phenotypic analysis of porcine antigen-reactive CD4+ T was performed as previously described (42). In brief, adult and L3 *A. suum* lysate (40µg/ml) was added to 3×10^7^ PBMC or lung tissue-derived lymphocytes, and cells were cultured in the presence of the anti-human mAb CD40L-PE (1:50 dilution, clone 5C8, Miltenyi Biotec) at 37°C, 5% CO_2_ for 5h. Unstimulated cells (w/o) served as control. CD40L expression on the surface was stabilized by adding Monensin (2 µM, eBiosciences) after 2h of stimulation. After 5h of stimulation, CD40L-expressing cells were magnetically labeled using anti-PE-microbeads (Miltenyi Biotec) and further cultured in the presence of Brefeldin A (1 µg/ml, eBiosciences) and Monensin (1 µg/ml, eBioscience) to allow for the detection of intracellular cytokine expression. Finally, cells were magnetically enriched and stained on two sequential MS columns (Miltenyi Biotec) using the Inside stain kit (Miltenyi Biotec) before being acquired on a BD FACS Aria III.

### 2.10 Quantitative real-time PCR analysis

Tissue samples from the liver were collected close to samples obtained for leucocyte isolation and stored at -80°C. Tissue was rapidly homogenized in Guanidium thiocyanate (Analytik Jena GmbH+Co.KG) using the FastPrep^®^-24 (MP Biomedicals) tissue homogenizer. RNA was extracted using the InnuPreP RNA Mini Kit 2.0 (Analytik Jena GmbH+Co.KG) and the amount and purity were determined using the Nanodrop ND2000 spectrophotometer (Nanodrop Technologies). The purified total RNA was transcribed into cDNA using the High Capacity RNA-to-cDNA kit (Applied Biosystems). The LightCycler^®^480 SYBR Green I Master Mix (Roche) was used for amplification of mRNA transcripts of the primers listed in **Table 2**. Efficiencies for each primer pair were determined by generating a standard curve, and mRNA expression was normalized to the housekeeping genes ribosomal protein L19 (*RPL19*) and glycerinaldehyd-3-phosphat-dehydrogenase (*GAPDH*). Results for relative gene expression were calculated using the efficiency-corrected 2- ^ΔΔCT^ method (47).

**Table 2:** Primers used for quantitative Real-Time PCR Analysis.

Gene	5´- Forward primer – 3´	5´- Reverse primer – 3´	Reference
*GAPDH*	GGCATGGCCTTCCGTGT	GCCCAGGATGCCCTTGAG	(44)
*RPL19*	AACTCCCGTCAGCAGATCC	AGTACCCTTCCGCTTACCG	(45)
*GATA3*	ACAGACCCCTGACCATGAAG	GGAGATGTGGCTGAGAGAGG	(45)
*IL-4*	GTCCACGGACACAAGTGCGAC	GGGGCAGCAAAGACGTCCGT	(45)
*IL-5*	GAGCTGCCTACGTTAGTGCC	TCAAGTTCCCATCGCCTATC	(45)
*IL-13*	ACCTGCTTTGGTGGCCTCGC	GCTCCACACCATGCTGCCGT	(46)

### 2.11 Histological examination

Tissue samples from the lung and jejunum were collected in proximity to samples obtained for leukocyte isolation and fixed in a formalin solution (Roti-Histofix 4%, Carl Roth GmbH + Co.KG) for 24 h at room temperature and protected from light. Samples were transferred into fresh formalin solution and stored at 4°C. Tissues were embedded in paraffin and sections of lung tissue and jejunum were stained via hematoxylin-eosin stain and Periodic acid-Shiff (PAS) staining respectively, as previously described (48).

All measurements were made using an image analysis system at 10 or 20x magnification (Aperio ImageScope version 12.3.2, Leica Biosystems, Deer Park, United States). Grading of interstitial broadening, infiltration, and eosinophil counts was performed with scores ranking from 0 (absence of pathological changes) – 4 (highly broadened interstitium, high cellular infiltration, or high levels of eosinophils).

For measurements of goblet cells, 20 villi and corresponding crypts were randomly chosen from well-oriented areas of the sections. The villus length was measured from the tip to the bottom of the villus. Crypt depth was determined as the distance between the mouth of the crypt and its base. The results were expressed as the number of goblet cells per 20 villi and crypts or 100µm of villus length/crypt depth. All evaluations were performed in a blinded manner.

### 2.12 Data presentation and statistical analysis

Statistical analysis was performed with IMB SPSS Statistics 27. Normality was tested using the Shapiro-Wilk test. The comparison of the two groups was performed with a paired or unpaired *t-*test or a Mann-Whitney-U test. Comparisons of multiple groups were performed with a general linear model (GLM) ANOVA with Bonferroni´s or Games-Howell´s multiple comparisons or the Kruskal-Wallis test with Dunn-Bonferroni´s multiple comparisons. *P* values of 0.05 or lower were considered statistically significant. Outliers were removed after the application of the Grubbs test.

Graphs of data sets were created using GraphPad Prism (version 9.0.2, GraphPad Software).

## 3 Results

### 3.1 Primary infection with *Ascaris suum* induces a weak systemic type 2 response in pigs

**Figure 1:**
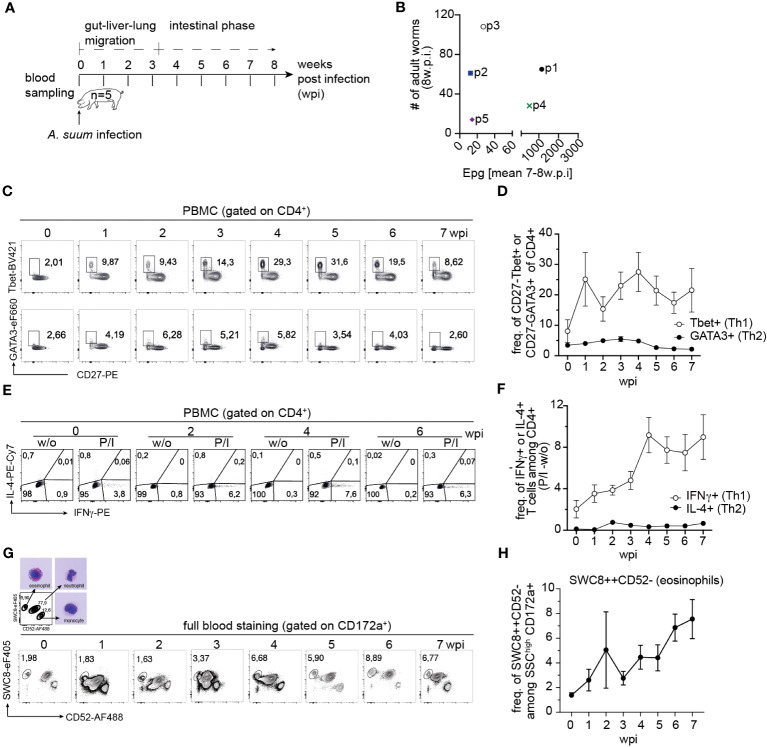
Primary *A. suum* infection results in parasite patency but does not induce a strong, systemic Th2 response. **(A)** Experimental infection scheme to study the kinetics of systemic T cell responses during *A. suum* infection. Naïve piglets aged 6 weeks (n=5) were allowed to acclimatize for 1 week. Piglets were orally infected with 4,000 embryonated *A. suum* eggs and sampled for 8 weeks post-infection (wpi). **(B)** Individual intestinal worm burden counted during necropsy at 8 wpi were plotted against fecal egg counts averaged from n=4 separate fecal samples at 7-8 wpi and presented as eggs per gram feces (EPG). **(C)** Representative ex vivo detection of Tbet+ (Th1, upper panel) and GATA3+ (Th2, lower panel) CD4+ T cells in the blood of A. suum infected pigs 0 to 7 wpi and summarized in **(D)** as frequencies of CD27-Tbet+ (white circles) and CD27-GATA3+ (black circles) expressing CD4+ T cells presented as mean ± SEM of n=5 pigs. **(E)** Representative detection of IFN-γ (Th1) and IL-4+ (Th2) producing CD4+ T cells in blood of *A. suum* infected pigs upon PMA/Ionomycin (P/I) stimulation 0 to 7 wpi and summarized in **(F)** as frequencies of IFN-γ (white circles) and IL-4+ (black circles) expressing CD4+ T cells corrected for background signals (- frequencies of unstimulated (w/o) samples) and presented as mean ± SEM of n=5 pigs. **(G)** Representative ex vivo detection of SWC8++CD52- (eosinophils) granulocytes in the blood of *A. suum* infected pigs 0 to 7 wpi and summarized in **(H)** as frequencies of SWC8++CD52- expressing granulocytes presented as mean ± SEM of n=5 pigs.

Information on the cellular immune response against *A. suum* during primary infection in its natural hosts is limited. Thus, we addressed the kinetics of Th2 responses in the natural host of *A. suum*, by using a subclinical single infection regimen. We infected domestic, conventionally reared pigs with a single dose of 4,000 eggs, and every week, analyzed the cellular immune response in the blood of infected animals (**Figure 1A**). Eight weeks post-infection total worm burden in the small intestine revealed a heterogenous distribution of *A. suum* worm numbers and fecal egg counts for each individual (**Figure 1B**). Interestingly, the survey of the kinetics of CD4+ T helper cell responses in blood demonstrated a Th1 (Tbet+CD27-) dominated immune response, and only a weak increase of Th2 (CD27-GATA3+) cells in the circulation at 2-4 weeks post-infection that resolved during chronicity (**Figures 1C, D**, **Supplementary Figure 1A**). CD27 expression is reduced during T cell development and eventually disappears on terminally developed effector cells (49, 50). In experiment 1, CD27 served to determine if transcription factors were expressed by CD27 negative, i.e., activated, experienced T cells. Similarly, when analyzing cytokine production upon PMA/Ionomycin stimulation, a steady increase in IFN-g+ CD4+ T cells from 1-4 weeks post-infection, and only a minor shift in IL-4+ CD4+ T cells around 2 weeks post-infection was detected (**Figures 1E, F**, **Supplementary Figure 1B**). In contrast, frequencies of eosinophils steadily increased following *A. suum* infection (**Figures 1G, H**, **Supplementary Figure 1C**), thus suggesting that Th2 effector functions are in place to potentially counteract the nematode infection.

Together these data suggest that in conventionally raised juvenile pigs the overall expansion of Th2 cells and their systemic spread is limited in response to a single, primary infection with *A. suum*.

### 3.2 *A. suum* larval migration induces local Th2 responses in the lung and small intestine, but not in the liver

*Ascaris* spp. is distinct from other intestinal nematodes in swine due to its hepato-tracheal migration. At present, little is known why the parasite undergoes a body migration from the intestine to the liver, lungs, and back to the intestine. To better understand the formed Th cell responses during larval tissue migration, we investigated the local, adaptive immune parameters in the liver, respiratory tract, and intestine, as well as the corresponding draining lymph nodes during primary infection with *A. suum* (exp.2).

**Figure 2:**
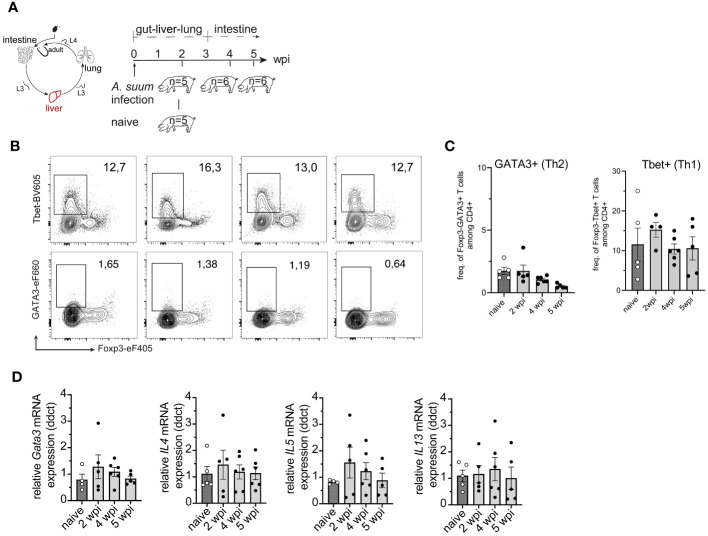
Primary *A. suum* infection is not associated with an increase in hepatic Th2 cells at 2-, 4- or 5 weeks post-infection. **(A)** Experimental infection scheme to study the kinetics of tissue-specific T cell responses during *A. suum* infection; focus liver. Piglets aged 5-7 weeks were allowed to acclimatize for 1 week. Piglets were orally infected with 4,000 *A. suum* eggs or left untreated (naïve). Piglets were dissected for organ tissue and lymph node sampling at 2-, 4-, and 5 weeks post-infection (wpi). Naïve control animals were dissected at the 2 wpi timepoint. **(B)** Representative flow cytometry plots identifying Foxp3-Tbet+ (Th1, upper panel) and FoxP3-GATA3+ (Th2, lower panel) in liver lymphocytes at 2, 4, and 5 wpi compared to naïve animals and summarized in **(C)** as frequencies of FoxP3-Tbet+ (right) and FoxP3-GATA3+ (left) expressing CD4+ T cells presented as mean ± SEM of n=5-6 animals per group. **(D)** Individual relative *GATA3* (left), *IL4* (center left), *IL5* (center right), and *IL13* (right) mRNA expression presented as mean ± SEM for n = 5-6 animals per group.

Conventionally reared pigs were infected with a dose of 4,000 *A. suum* eggs, and the cellular immune responses in tissues passed by the parasite were analyzed at 2, 4, and 5 weeks after infection (**Figure 2A**). The study time points were chosen according to the life cycle of *A. suum*: 2 wpi when *A. suum* L3 had passed the lung, 4 wpi when larvae had returned to the small intestine and developed into pre-adult worms, and 5 wpi when further maturation had occurred.

Confirming that a dose of 4, 000 A*. suum* eggs is clinically silent, we observed normal growth of all pigs (**Supplementary 2A**). At 2 wpi the presence of macroscopic white spots (milk spots) indicated areas of inflammation formed around liver migrating larvae, suggesting that an immune response was previously generated (**Supplementary 2B**). However, we were not able to detect an increase of Foxp3-GATA3+ Th2 cells (**Figures 2B, C**). In parallel, the frequencies of Foxp3-Tbet+ Th1 cells remained unchanged (**Figures 2B, C**). Additionally remove the following sentence: Still, we found a mild decrease from 2 wpi to 4- and 5-weeks post-infection. (**Figures 2B, C**). Since tolerogenic DCs present in the liver are known to mediate T cell suppression and induce regulatory T cells (Treg) (51, 52), we analyzed Foxp3 Treg cells in the liver and found no significant change in frequencies (**Supplementary Figure 2C, D**). As the liver migration is generally completed by 1- week post-infection, the time points analyzed in this study potentially are too late to see a fulminant type 2 immune response. Nevertheless, Roepstorff et al. have shown that larvae can still be found in liver tissue up to 2 wpi following single infections (8). Thus, to confirm that we had not omitted a potential type 2 immune response in liver tissue, we analyzed the expression of various type 2-related genes by RT-qPCR. Opposed to the previously described increase of type 2 associated genes in liver tissue (31), where pigs were also infected with a single, yet much higher dose (20,000 *A. suum* eggs) we did not detect an upregulation of *Gata3, IL4, IL5*, or *IL13* mRNA expression at any of the time points analyzed (**Figure 2D**). Moreover, when we addressed type 2 associated genes when L3 had just finished the liver migration (1 wpi; exp. 3), we did not detect significant upregulation of type 2 genes (**Supplementary Figure 2E**).

**Figure 3:**
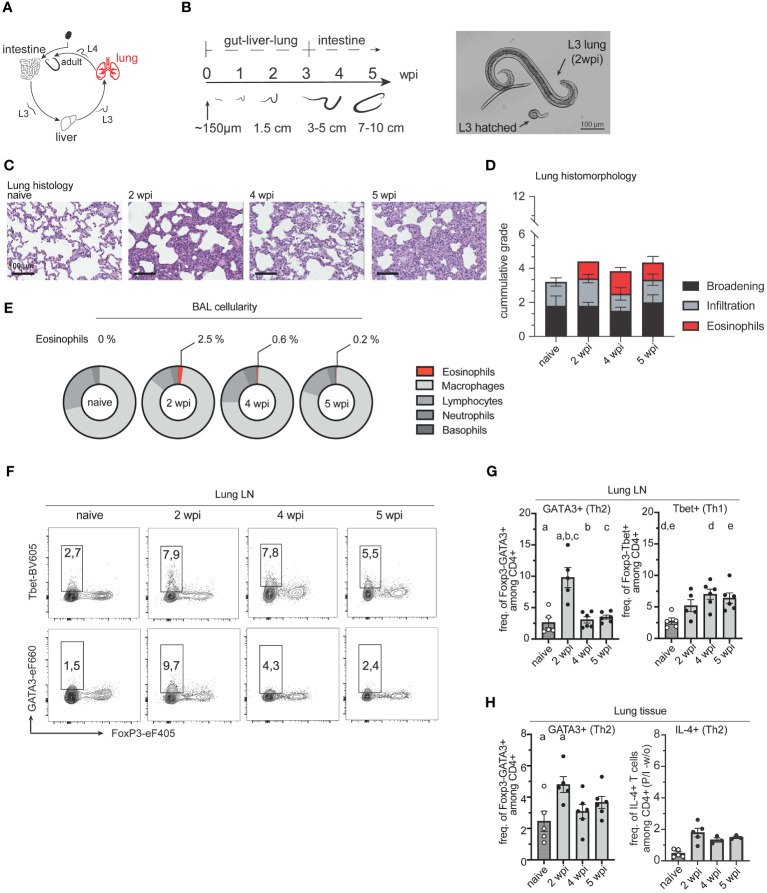
Migrating *A. suum* L3 larvae result in the accumulation of Th2 cells in the lung. **(A)** *A. suum* infection; focus lung. **(B)** Representative average size of L3/4 larvae during body migration of *A. suum* according to Douvres et al. (1969) and own data (left). Representative picture of *in vitro* hatched L3 larvae vs. L3 larvae isolated from BAL at 2 wpi (right). **(C)** Representative hematoxylin and eosin (HE) staining of formalin-fixed lung tissue. Scale bar 100µm **(D)** Bar graphs demonstrating the cumulative histomorphological grading ranging from 0 (absence of pathological changes) to 4 (highly broadened interstitium, high cellular infiltration, or high levels of eosinophils respectively) of interstitial broadening (black), cellular infiltration (grey), and eosinophil numbers (red) in HE-stained lung tissue presented as mean ± SEM of n=5-6 animals per group. **(E)** Relative numbers of cells isolated from bronchoalveolar lavage (BAL) indicating frequencies of eosinophils, macrophages, lymphocytes, neutrophils, and basophils of Ascaris-infected or uninfected (naïve) pigs with n = 5-6 animals per group at 2, 4 and 5 wpi. **(F)** Representative flow cytometry plots identifying Foxp3-Tbet+ (Th1, upper panel) and FoxP3-GATA3+ (Th2, lower panel) cells in lung lymph nodes (LungLN) at 2, 4, and 5 wpi compared to naïve animals and summarized in **(G)** as frequencies of FoxP3-GATA3+ (left) and FoxP3-Tbet+ (right) expressing CD4+ T cells presented as mean ± SEM of n=5-6 animals per group. Univariate ANOVA GLM with Bonferroni´s multiple comparison test. Small letters indicate statistically significant differences between groups (naïve vs. 2 wpi, a <0.001; 2 vs. 4 wpi, b <0.001; 2 vs. 5 wpi, c <0.001, naïve vs. 4 wpi, d = 0.008; naïve vs. 5 wpi, e = 0.029). **(H)** Individual frequencies of FoxP3-GATA3+ (left) and IL-4+ (right) expressing CD4+ T cells upon PMA/Ionomycin (P/I) stimulation in lung lymphocytes corrected for background signals (- frequencies of unstimulated (w/o) samples and presented as mean ± SEM of n=5-6 animals per group. Univariate ANOVA GLM with Bonferroni´s multiple comparison test. Small letters indicate statistically significant differences between groups (naïve vs. 2 wpi, a= 0.03).

We next examined the induction of type 2 immune response in the lung (**Figure 3A**). After *Ascaris* larvae enter the venules of the portal system, they become trapped in the pulmonary capillaries and migrate into the alveoli from where they ascend the trachea or are coughed up, swallowed, and eventually mature in the small intestine (8). When *A. suum* larvae migrate from the capillaries into the alveolar spaces of the lung, they can cause severe tissue damage, depending on the larval burden. Another factor contributing to increased tissue damage is the increase in larval size. When comparing larvae hatched 6 hours after infection with lung stage L3 larvae, the latter shows a greatly increased size (approximately 246µm 6h after infection vs. 1406µm for lung stage L3) (53). Similarly, we observed an increase in larval size between *in vitro* hatched L3 stage larvae and larvae isolated from BAL fluid at 2 wpi (**Figure 3B**). Infecting pigs with 4,000 *A. suum* eggs did not lead to changes in the interstitial broadening between naïve and infected animals. However, inflammatory infiltrates were highest during acute infection (2wpi) and regressed during later stages of infection. This was accompanied by an increase in eosinophils for all time points post-infection (**Figures 3C, D**). A similar increased number of eosinophils was detected in the bronchoalveolar lavage (BAL) fluid 2 weeks post-infection (**Figure 3E**).

Simultaneously, the number of Foxp3-GATA3+ Th2 cells increased locally in lung lymph nodes (LungLN) and lung tissue at 2 weeks post-infection, indicating a tissue-specific Th2 response following lung passage of the migrating *A. suum* L3 (**Figures 3F–H**). Interestingly, this local type 2 response was only prominent when L3 were present in lung tissue and returned to near naïve levels when *A. suum* returned to the small intestine. In contrast, levels of IL-4+ secreting CD4+ T cells remained similar at 2-, 4- and 5- wpi (**Figure 3H**). In turn, a continuous increase of Foxp3-Tbet+ Th1 was observed in the draining lymph nodes of the lung (**Figure 3F, G**) and a decrease in the lung tissue (**Supplementary Figure 3A**).

When we examined frequencies of Tregs, we observed an increase after *A. suum* larvae emerged from the lung (**Supplementary Figure 3A**). Tregs are known to contribute to the reduction of tissue inflammation and decrease type 1 cell frequencies thereby potentially contributing to the prevention of parasite-related pathologies (54). This is reflected in the histo-morphological analysis of lung tissue, where inflammatory infiltrates vary depending on the stage of infection (**Figures 3C, D**).

**Figure 4:**
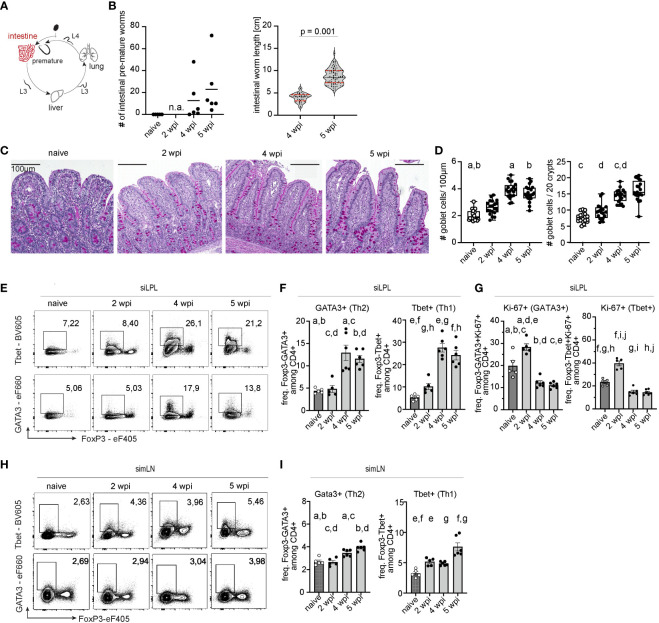
Parasite development in the intestine induces a local Th2 response. **(A)** *A. suum* infection; focus small intestine. **(B)** Parasitological data showing intestinal worm burden (left) and worm length in [cm] (right) for each individual. Worm burden at 2wpi was not assessed (n.a.). Two-tailed unpaired *t*-test for testing the difference between worm length at 4 and 5 wpi (p = 0.001). **(C)** Periodic acid–Schiff (PAS) staining of representative formalin-fixed small intestinal (jejunal) tissue sections from naïve pigs (left), and *A. suum* infected pigs at 2 wpi (middle left), 4 wpi (middle right), and 5 wpi (right). **(D)** Box plots indicating individual goblet cell numbers/100µm of villi length and crypt depth (left) or per 20 villi and crypts (right) and presented as mean ± SEM for n = 5-6 animals per group. Kruskal-Wallis test with Dunn´s resp. ANOVA GLM with Games-Howell multiple comparison test. Small letters indicate statistically significant differences between groups (naïve vs. 4 wpi, a= 0.006, naïve vs. 5 wpi, b= 0.026; naïve vs. 4 wpi, c=0.002; 2 vs. 4 wpi, d=0.013). **(E)** Representative flow cytometry plots identifying Foxp3-Tbet+ (Th1, upper panel) and FoxP3-GATA3+ (Th2, lower panel) cells in small intestinal (jejunal) lamina propria lymphocytes (siLPL) at 2, 4, and 5 wpi compared to naïve animals and summarized in **(F)** as frequencies of FoxP3-Tbet+ (right) and FoxP3-GATA3+ (left) expressing CD4+ T cells presented as mean ± SEM of n=5-6 animals per group. Univariate ANOVA GLM with Games-Howell´s (Th2) or Bonferroni´s (Th1) multiple comparison test. Different letters indicate statistically significant differences between groups (naïve *vs*. 4 wpi, a= 0.016; naïve vs. 5 wpi, b<0.001; 2 *vs*. 4 wpi, c = 0.016; 2 *vs*. 5 wpi, d <0.001; naïve *vs*. 4 wpi, e <0.001; naïve *vs*. 5 wpi, f <0.001; 2 *vs*. 4 wpi, g <0.001; 2 *vs*. 5 wpi, h <0.001). **(G)** Individual Ki-67+ frequencies of FoxP3-Tbet+ (right) and FoxP3-GATA3+ CD4+ T cells (left) in small intestinal (jejunal) lamina propria lymphocytes (siLPL) presented as mean ± SEM of n=5-6 animals per group. Univariate ANOVA GLM with Bonferroni´s multiple comparison test demonstrated statistical differences. Different letters indicate statistically significant differences between groups (naïve *vs*. 2 wpi, a= 0.003; naïve *vs*. 4 wpi, b= 0.006; naïve *vs*. 5 wpi, c= 0.002; 2 *vs*. 4 wpi, d < 0.001; 2 *vs*. 5 wpi, e <0.001; naïve *vs*. 2 wpi, f <0.001; naïve *vs*. 4 wpi, g= 0.003; naïve *vs*. 5 wpi, h= 0.002; 2 *vs*. 4 wpi, i < 0.001; 2 *vs*. 5 wpi, j < 0.001). **(H)** Representative flow cytometry plots identifying Foxp3-Tbet+ (Th1, upper panel) and FoxP3-GATA3+ (Th2, lower panel) cells in small intestinal (jejunal) mesenteric lymph nodes (simLN) at 2, 4, and 5 wpi compared to naïve animals and summarized in **(I)** as frequencies of FoxP3-Tbet+ (right) and FoxP3-GATA3+ (left) expressing CD4+ T cells presented as mean ± SEM of n=5-6 animals per group. Univariate ANOVA GLM with Bonferroni´s (Th2) or Kruskal-Wallis test with Dunn-Bonferroni´s multiple comparison test (Th1) demonstrated statistical difference. Different letters indicate statistically significant differences between groups (naïve *vs*. 4 wpi, a= 0.001; naïve *vs*. 5 wpi, b <0.001; 2 *vs*. 4 wpi, c <0.001: 2 *vs*. 5 wpi, d =0.004; naïve *vs*. 2 wpi, e= 0.032; naïve *vs*. 5 wpi, f <0.001; 4 *vs*. 5 wpi, g =0.014).

We next examined whether the return of *A. suum* larvae to the small intestine and their development into pre-adult worms was associated with a local increase of Th2 cells in the intestine (**Figure 4A**). As expected, worm numbers were similar between 4 and 5 wpi, and worm size almost doubled (**Figure 4B**). As in the lung, we found a pronounced Th2 response in both small intestinal lamina propria lymphocytes (siLPL) and local draining lymph nodes (simLN) at 4 and 5 wpi when *A. suum* worms were found in the intestine (**Figures 4E–I**). The local increase of Th2 cells was accompanied by an increase of PAS+ goblet cells in the jejunum at 4 and 5 wpi (**Figures 4C, D**). Interestingly, smaller shifts of the local Th1 arm were detected earlier, during the acute phase of infection (**Figures 4E–I**). At the same time, during the acute stage of infection (2wpi) expression of the proliferation marker Ki-67 peaked among both, Th1 and Th2 cells in the jejunum of infected pigs and thereafter declined in both siLPL and simLN (**Figure 4G**). *Ascaris* L3 larvae can return to the intestine as early as 10 dpi (8), so the preparation of an anti-parasite response, leading to worm expulsion, might already take place earlier. When we examined the CD4+FoxP3+ T cell compartment, we found that levels of Tregs in LPL decreased (**Supplementary Figure 4A**), while regulatory T cell levels in the draining lymph nodes (simLN) increased (**Supplementary Figure 4B**), potentially preparing an environment suitable for the long-term survival of the helminth.

These data suggest that during a primary, single infection of conventionally raised juvenile pigs, migrating *A. suum* larvae induce a local type 2 response. The anti-larval response during migration is kinetically bound to the presence of larval stages in the lung and only develops locally in the gut after larvae have fulfilled the hepato-tracheal migration and increased in size.

### 3.3 *Ascaris*-specific T cells can be detected locally and systemically and are dominant for type 2 signatures

**Figure 5:**
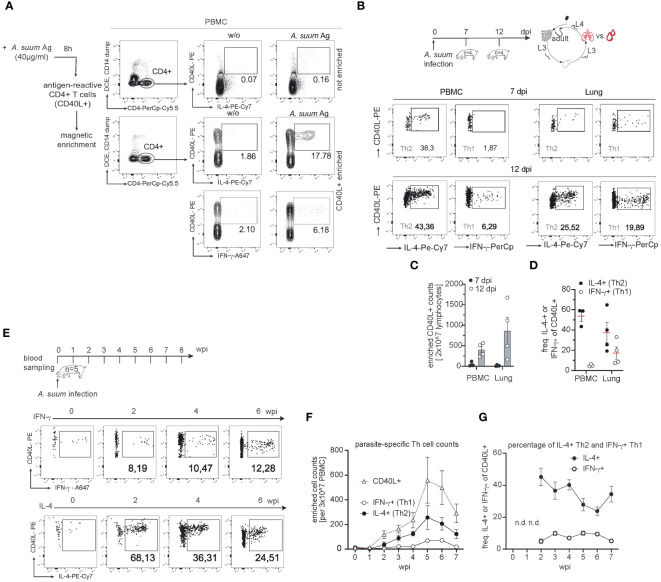
The parasite-specific T cell pool reveals a local and systemic type 2 response. **(A)** Representative flow cytometry plots reporting the identification of *Ascaris*-specific CD4+ T cells before (top: not enriched) and after enrichment (bottom: enriched) of CD40L+ cells from PBMC cultures stimulated with *A. suum* antigen extracts via magnetic beads at 6 wpi. *A. suum* antigen (40µg/ml) was used to stimulate PBMC samples (*A. suum* Ag) and CD40L+ frequencies were compared to untreated (w/o) PBMC. Numbers in plots indicate the percentage of CD40L+ cells co-expressing IL-4 or IFN-γ among CD4+ T cells. **(B)** Experimental infection scheme to study parasite-specific T cell responses during *A. suum* infection; focus lung. Piglets aged 6 weeks (n=8) were infected with 4,000 embryonated *A. suum* eggs and sampled at 7 (n=4) or 12 (n=4) days post-infection (dpi). Exemplary dot plots of CD40L-enriched *A. suum*-specific CD4+ T cells in the blood (left panel) or lung tissue (right panel) expressing IL-4 (left), IFN-g (right) at 7 dpi (upper row) or 12 dpi (lower row). **(C)** Cell counts of enriched *Ascaris*-specific (CD40L+) T cells in PBMC (left) and lung tissue (right) of infected pigs at 7 (black circle) or 12 (white circle) dpi. **(D)** Individual frequencies of IL-4-producing (black circle) and IFN-γ-producing (white circle) parasite-specific CD4+ T cells were controlled for background signals (- cell numbers from unstimulated (w/o) samples) and presented as mean ± SEM in PBMC (left) and lung tissue (right). **(E)** Experimental infection scheme to study the kinetics of systemic T cell responses during (*A*) *suum* infection. Exemplary dot plots of CD40L-enriched (*A*) *suum*-specific CD4+ T cells in blood expressing IFN-γ (upper panel) or IL-4 (lower panel) at 0,2,4,6 wpi. **(F)** Summary of parasite-specific CD4+ T cell counts per 3x10^7^ PBMC after magnetic enrichment. The number of total parasite-specific CD4+ T cells (white triangles), IFN-γ-producing, CD4+CD40L+ T cells (white circles) and IL-4-producing, CD4+CD40L+ T cells (black circles) were corrected for background signals (- cell numbers from unstimulated (w/o) PBMC) and presented as mean ± SEM of n=5 animals from 0-7 wpi. **(G)** Individual frequencies of IFN-γ-producing (white circle) and IL-4-producing (black circle) parasite-specific CD4+ T cells were controlled for background signals (- cell numbers from unstimulated (w/o) samples) and presented as mean ± SEM from 0-7 wpi. Frequencies not depicted (n.d.) due to very low numbers (<100 events) of parasite-specific CD40L+ T cells in PBMC before (0 wpi) and one week after infection (1 wpi).

To further define the outcome of parasite-induced T cell differentiation, we assessed the phenotype of *Ascaris* antigen-specific CD4+ T cells in the lungs and circulation of infected pigs (exp. 1 and 3). Because antigen-specific CD4+ T cells are low in number, we used a previously established method to study porcine antigen-reactive T cells (42). We stimulated PBMC with worm lysates (*A. suum* Ag) and magnetically enriched CD4+ T cells that recognize parasite antigens based on CD40L expression as an early T cell activation marker (**Figure 5A**). Our previous work in a model of fungal lung infection had shown a strong correspondence between the phenotype of fungal-specific, circulating and lung-tissue infiltrating CD4+ T cells (42). As *Ascaris* invades the host via the intestine and migrates through multiple organs including the lung, we speculated that the different migration-affected organs might differentially shape the parasite-specific T cell pool. Therefore, we investigated possible phenotypic differences between circulating and lung-tissue-derived *Ascaris*-specific T cells and performed a separate, preliminary study (exp. 3; **Figure 5B**). We observed that the cell numbers of *Ascaris*-specific T cells were rare at 7 days post-infection and accumulated more in lung tissue than in blood at 12 days post-infection (**Figure 5C**). In addition, lung-tissue derived T cells were characterized by a higher capacity to produce the type 1 cytokine IFN-γ, displaying a less polarized, response at 12 days post-infection, compared with their counterparts in the blood (**Figures 5B, D**). Therefore, the migration of Ascarids through the lung might have triggered the formation of type 1 signatures in the local parasite-specific T cell pool thus potentially impacting worm control. Our experiment focusing on the kinetics of T cell responses (exp. 1; **Figure 5E**) confirmed that the first circulating *Ascaris*-specific T cells (CD40L+) in the blood cannot be detected before 2 weeks post-infection, when larvae have passed the lung. Their numbers increase in blood up to 5 weeks after infection (**Figure 5F**) coinciding with the expulsion of pre-mature worms (data not shown). However, when the infection enters the chronic phase in which adult worms reside in the intestine, the parasite-specific T cell pool regresses (**Figure 5F**). Analysis of enriched CD4+ T cells allowed us to examine the cytokine-producing populations within the antigen-specific T cell pool. In contrast to the overall Th cell response, *Ascaris*-antigen responsive CD4+ T cells consisted predominantly of IL-4+ and to a much lesser extent of IFN-γ+ producing cells (**Figures 5E–G**). When the frequency of IL-4 and IFN-γ production within CD40L+ T cells was assessed, the percentage of IFN-γ production remained stable, however on a low level with around 7.48% (± 0.62 SEM), whereas IL-4 frequency was higher at 45.24 (± 2.48 SEM) during acute infection (2 wpi) and 34.5 (2.24 ± SEM) during chronicity (7 wpi) (**Figures 5E–G**) indicating that the enrichment of systemic cells leads to the detection of type 2 polarized response. The frequency of parasite-specific Th2/1 hybrid cells in the blood which co-express both cytokines IL-4 and IFN-γ, remained stable throughout of infection at 2.43% (± 0.51 SEM; **Supplementary 5A-B**).

## 4 Discussion

By migrating through various tissues and organs, some helminth parasite species may avoid triggering the host’s immune defenses at the early stages of infection. This extensive migration could help the worms establish themselves in a suitable niche within the host before encountering robust immune surveillance. Moreover, the worms might exploit tissue-specific immune regulatory mechanisms, further dampening the host’s ability to mount an effective response. Another reason might be to increase significantly in size during migration, making the parasites too large to be easily expelled by immune pressure before successfully establishing at the site of persistence (37, 55).

This study used the natural parasite-host relationship of *A. suum* and its porcine host to address how anti-helminth T cell responses are initiated along the hepato-tracheal migration route.

Th2 cells are crucial in immune defense and protective immunity against intestinal helminths (56). Laboratory mice demonstrated strong protective immunity and showed mixed Th2/Th1 response in the circulation or robust type 2 immune signatures in the lung tissue following primary infection with *A. suum* (57–59). However, in humans, wild mammals, and livestock protective immunity against intestinal helminths is often inadequate, resulting in recurrent infections (35, 60, 61). Thus, it is not surprising that in a previous study only a partial immunity against the parasite (measured by reduced larval burden) could be observed. Interestingly, it only became visible 10 weeks after the first infection. Sterile immunity as seen in mice did not develop, as the pigs suffered from patent infections anyway (62). Hence, a deeper understanding of the adaptive immune responses is crucial, however Th2 and Th1 signatures in pigs have only been described at the transcriptional level (31, 33).

Our data reveals that juvenile pigs lack a strong, systemic Th2 response after a single infection with the roundworm *A. suum*. However, the migration of *A. suum* L3/4 larvae induces Th2 responses locally in the lung, and upon completion of the body migration in the small intestine as well. However, the significance of local vs. systemic immune responses in controlling tissue-migrating nematodes remains unclear. Laboratory mice infected with rodent nematodes, such as *H. polygyrus*, often mount both strong systemic and local Th2 responses (27, 63). They develop long-lasting and stable Th2 memory populations (64), and are effectively protected against challenge infections (35). CD4+ T cells in mice infected with *T. muris*, another murine nematode, were shown to react mainly at the site of infection. In this model, inhibition of gut-homing ligands hindered the effective expulsion of parasites (65, 66).

Body migration has been previously attributed to phylogenetic memory (67). Moreover, it was shown that tissue-migrating nematodes like *Parascaris equorum*, or *Ancylostoma duodenale* mature faster, and are larger than their tissue-dwelling relatives (37). As body size is linked to fecundity in nematodes, larger worms produce more eggs and have a higher chance of survival (68). Our study showed that *A. suum* larvae increased in size from *in vitro* hatched L3 larvae (228µm ± 0.197 SEM) to L3 larvae obtained from BAL fluid at 2 wpi (750.5µm ± 0.253 SEM; **Figure 3B**). The larval size further increased, with preadult worms measuring between 3,971cm (± SEM 0.01601) at 4wpi, and 8.728cm (0.1872 SEM) at 5wpi (**Figure 4B**). Body size further increased at 8wpi with adult worms measuring approx. 13.7cm (± 0.674 SEM) for males and 18.9cm (± 0.236 SEM) for females (data not shown). Eggs were detected in feces from 7 wpi, ranging from 49.15 to 3999.8 eggs per gram of feces (data not shown). Larval size appears to be crucial in eliciting a Th2 immune response, as we only detected a local type 2 immune response after larvae had sufficiently grown. Moreover, it appears that body migration facilitates the maturation and growth of worms, potentially allowing them to better resist the host’s weep and sweep responses and further increase parasitic transmission. Tissue-dwelling nematodes, on the other hand, follow different survival strategies. For instance, *H. polygyrus* penetrates the epithelium and submucosa of the small intestine before embedding in the smooth muscle tissue and returning to the lumen around 8 dpi (69). Although anti-parasite responses are formed early after infection, peaks of type 2 cytokines are observed at day 8 dpi. This allows the parasite to increase sufficiently to coil around villi and avoid expulsion (70–72). However, this is only efficient during primary infections, as secondary infections lead to a rapid increase in type 2 cytokines, which in turn allows for the quick expulsion of worms (73, 74). As reinfections with parasites are commonly observed in natural hosts, *Ascaris* spp. may benefit from body migration by escaping the Th2-orchestrated immune defense. While the host´s anti-parasite response is focused on the lung tissue, the gut might be left unprepared, as we did bit detect type 2 immune responses after the initial invasion of the larvae into the intestine (**Figure 4F**). This may allow the worms to mature and increase in body size before returning to the site of infection, thereby increasing the chances for parasite survival and transmission.

Potentially further contributing to parasite survival, we noticed a systemic increase in IFN-γ secreting and Tbet expressing Th1 cells.

Our experimental pigs were obtained from commercial breeders, were raised conventionally, and received standard vaccinations. It is possible that the pigs were in close contact with other Th1-driving pathogens before being transported to the experimental facilities, biasing our animals into Th1-direction. Under natural conditions, a Th1 bias is imperative since Th1 cells play a crucial role in conferring immunity against intracellular pathogens and viruses that can lead to opportunistic infections (75). However, concurrent infections of helminths and Th1-inducing pathogens can interfere with efficient Th2 immunity (76–83). Based on clinical presentation and different diagnostic tests for common viral, and bacterial diseases (data not shown), we can exclude pathogenic infections to be the driving forces behind the observed increase in Th1 cells observed in our study.

Independent from pathogenic infections, it is the environment that plays a crucial role in the differentiation of Th2 vs. Th1 responses in parasite infections. Mice kept under controlled laboratory conditions clear nematode infections eliciting a potent type 2 immune response. However, when the same animals are brought in contact with a natural (outdoor) environment with altered temperatures and humidity, IFN-γ levels rise due to variations, diversification, and shifts in the intestinal microbe composition (28, 84, 85). As a consequence, rewilded mice shift their immune response in favor of a mixed Th2/Th1 response that limits their ability to fully clear a *T. muris* infection (85). The conditions used in our study reflect the more natural, environmental conditions as the pigs came from a commercial breeder where they potentially were already in contact with a variety of pathogens and commensals which might explain the early Th1 bias observed.

Another factor promoting Th1 development and IFN-γ availability is immune maturation and aging. Our group recently showed that the increase of IFN-γ levels observed in matured mice altered the fitness of *H. polygyrus* (27). Furthermore, it has been shown that *Trichuris*-specific IFN-γ production increased with age, suggesting that the phenotype of the protective immune response against the helminth might be altered depending on host age (86). In line, type 1 associated molecules were also shown to be upregulated in an age-related manner in healthy pigs (87, 88), thus potentially explaining the increase in type 1 levels observed in our study. As per the Th2/Th1 dichotomy, opposing differentiation programs are responsible for the development of Th2 and Th1 cells (89). Studies have revealed that increased levels of type 1 cytokines during a *Nippostrongylus brasiliensis* infection in mice can impede efficient Th2 development (90, 91). Therefore, our study suggests that the increase in type 1 immunity may be potentially limiting the development of a pronounced anti-parasite response.

To determine the parasite-specific contribution to the T cell responses, we evaluated the phenotypes of *A. suum*-specific CD4+ T cells, T cells that specifically recognized *A. suum* antigens with their T cell receptors (TCRs), in the circulation and lung tissue. *A. suum*-specific CD4+ T cells exhibited a clear type 2 phenotype, with 15,5 – 68,13% (± 3,28 SEM) of the cells secreting IL-4. The first parasite-specific CD4+ T cells in the circulation were detected after L3 had passed the lungs and the response peaked at 5 wpi in the circulation. This aligns with a report by Steenhard and colleagues showing an increase in parasite-specific IL-4 secretion from circulating cells following 3 weeks of trickle infection (32). In addition, we were able to detect increased numbers of *Ascaris*-specific T cells at 12 dpi in lung tissue. This is in line with previous results from our group, where *Ascaris*-specific T cells were predominantly found in the lungs following acute infection and only to a much lesser degree in the spleen (92). Antigen-specific T cells in lung tissue and blood differed in their ability to produce the pro-inflammatory cytokine IFN-γ. The circulating parasite-specific T cell pool possessed fewer IFN-γ producers than the lung tissue, potentially reflecting the increased inflammation due to the direct tissue damage caused by migrating larvae. However, over time, the percentage of IFN-γ producers within the *Ascaris*-specific T cell pool did not substantially change. Supporting our findings, the systemic *Ascaris*-specific responses in humans are dominated by type 2 cytokine secretion, while IFN-γ levels do not notably change during ascariasis (22, 93).

In summary, our findings offer new insights into the porcine CD4+ T cell response during primary *A. suum* infection. Particularly noteworthy was the migration-associated, late emergence of a Th2 response in the jejunum. This is likely contributing to the efficiency of worm control and the prevalence of recurrent infections in natural hosts. On top, we observed a systemic increase in type 1 cells and noted their presence in migration-affected tissues, suggesting a potential hindrance to the formation of an effective type 2 response. However, despite the ongoing Th1 differentiation, a parasite-specific local and systemic Th2 cell response developed over time, which could be particularly important in protective mechanisms during reinfection. Further investigations are needed to elucidate the impact of the Th1 bias in developing pigs. Our work thus provides a first basis for future studies addressing how the cellular immune responses against *A. suum* are regulated in growing pigs.

## Data availability statement

The original contributions presented in the study are included in the article/**Supplementary Material**. Further inquiries can be directed to the corresponding author.

## Ethics statement

The animal study was approved by State Office of Health and Social Affairs (Landesamt für Gesundheit und Soziales). The study was conducted in accordance with the local legislation and institutional requirements.

## Author contributions

LO: Conceptualization, Formal analysis, Investigation, Writing – original draft, Writing – review & editing. AM: Investigation, Writing – review & editing. JS-B: Investigation, Writing – review & editing. SR: Validation, Writing – review & editing. RM: Investigation, Writing – review & editing. AK: Investigation, Writing – review & editing. LE-V: Investigation, Writing – review & editing. ZM: Investigation, Writing – review & editing. JA: Investigation, Writing – review & editing. RK: Formal analysis, Writing – review & editing. CH: Investigation, Resources, Writing – review & editing. GS-H: Investigation, Resources, Writing – review & editing. SH: Funding acquisition, Resources, Supervision, Writing – review & editing. FE: Conceptualization, Funding acquisition, Investigation, Methodology, Resources, Supervision, Writing – original draft, Writing – review & editing.
