# Supplementary material for: Ascaris suum infection in juvenile pigs elicits a local Th2 response in a setting of ongoing Th1 expansion
Source: Front Immunol. 2024 May 10;15:1396446. doi: 10.3389/fimmu.2024.1396446 (PMC11116563; doi:10.3389/fimmu.2024.1396446)
Supplement: Supplementary file 1 [file DataSheet_1.docx]

Supplementary Material

# Supplementary Figures and Tables

## Supplementary Figures


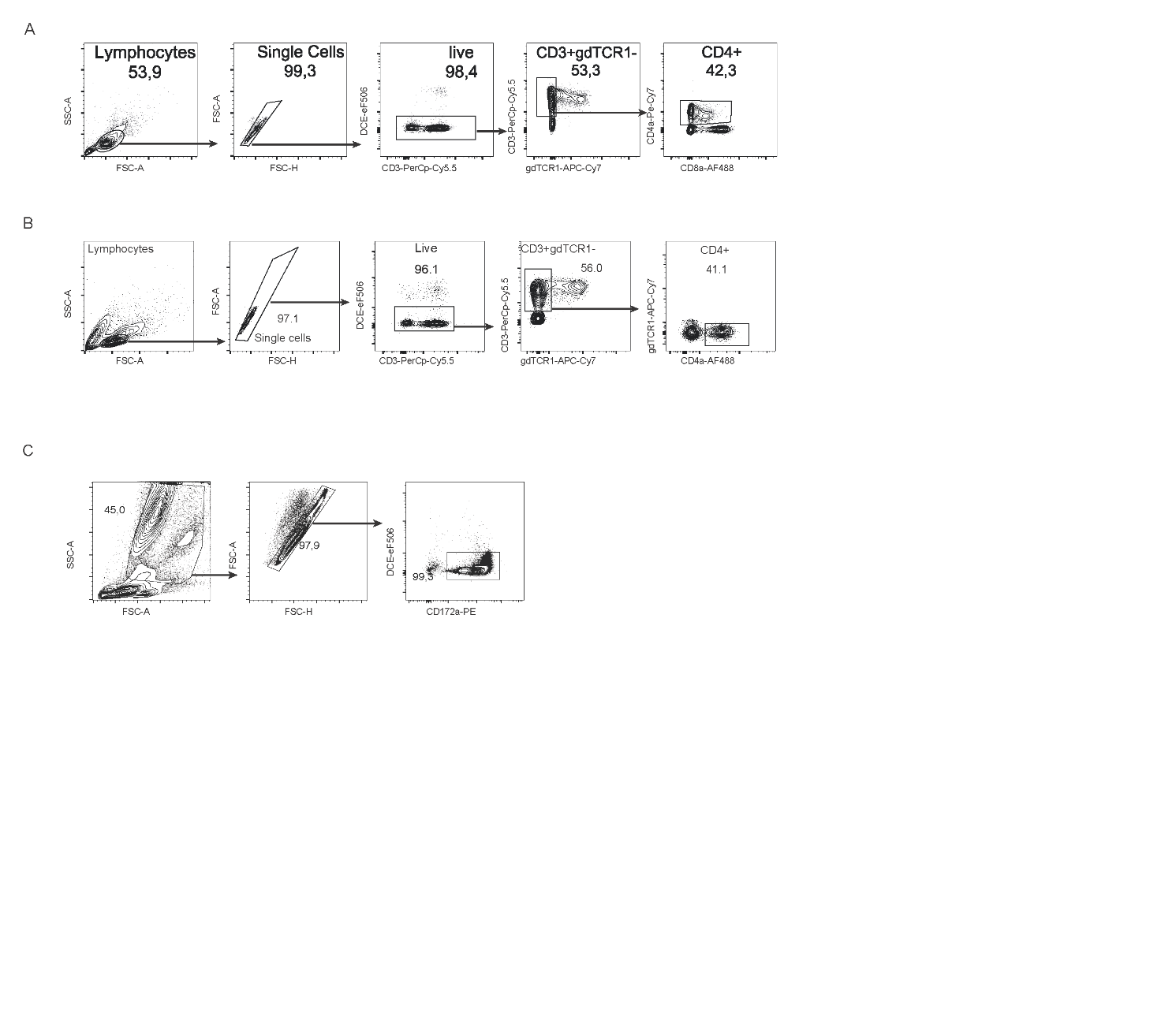


**Suppl. 1. Gating strategy for *ex vivo* detection of CD4+ T cells and eosinophils in blood**

A-B) Representative staining of PBMC for one animal. Lymphocytes were identified based on their light scatter properties (SSC-A *vs.* FSC-A), followed by doublets (FSC-A *vs.* FSC-H) and dead cell exclusion (viability dye negative cells). T cells were identified as CD3+gdTCR1- and further characterized based on their total CD4+ expression. C) Representative staining of whole blood for one animal. Granulocytes were identified based in their light scatter properties (SSC-A *vs.* FSC-A), followed by doublets (FSC-A *vs.* FSC-H), and dead cell exclusion (viability dye negative cells). Eosinophils were identified as CD172a+ cells and further characterized based on their SWC8 and CD52 expression as SWC8^high^CD52- cells.


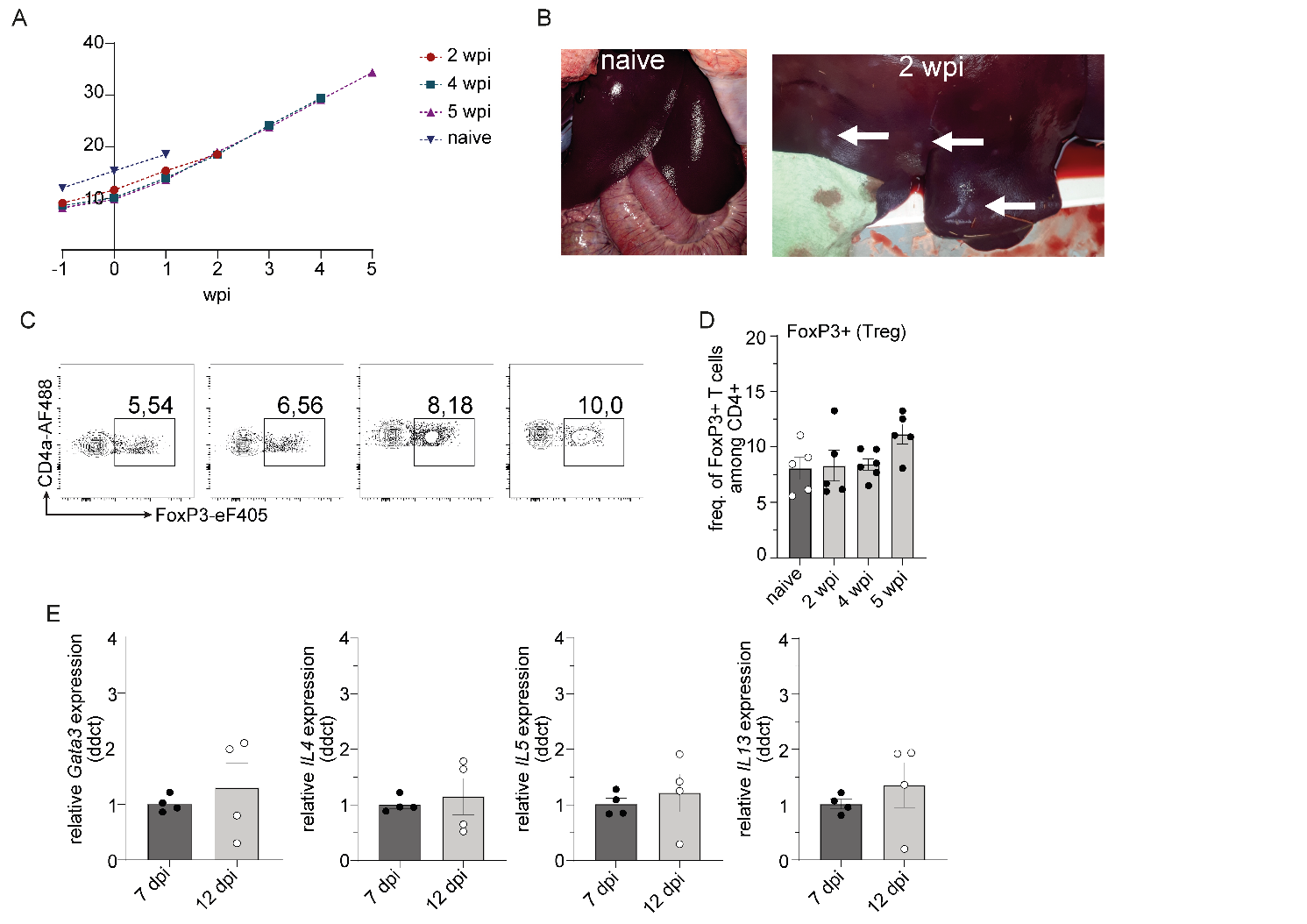


**Suppl. 2. Weight development, milk spots, hepatic Tregs, and hepatic type-2 associated genes in pigs.** A) Mean weight development of n= 5-6 pigs/ group after arrival (-1 wpi) and over the sampling period of 5 weeks post infection. B) Exemplary macroscopical liver white spot formation caused by cellular infiltrates of naïve (left) and infected (2 wpi; right) animals. C) Representative flow cytometry plots identifying Foxp3+ (Treg) cells in liver tissue at 2, 4, and 5 wpi compared to naïve animals and summarized in D) as frequencies of FoxP3+ expressing CD4+ T cells presented as mean ± SEM of n=5-6 animals per group. Univariate ANOVA GLM with Bonferroni´s multiple comparison test. E) Individual relative *GATA3* (left), *IL4* (center left), *IL5* (center right), and *IL13* (right) mRNA expression at 7 resp. 12 dpi (exp. 3) presented as mean ± SEM for n = 4 animals per group. Two-tailed unpaired *t*-test.


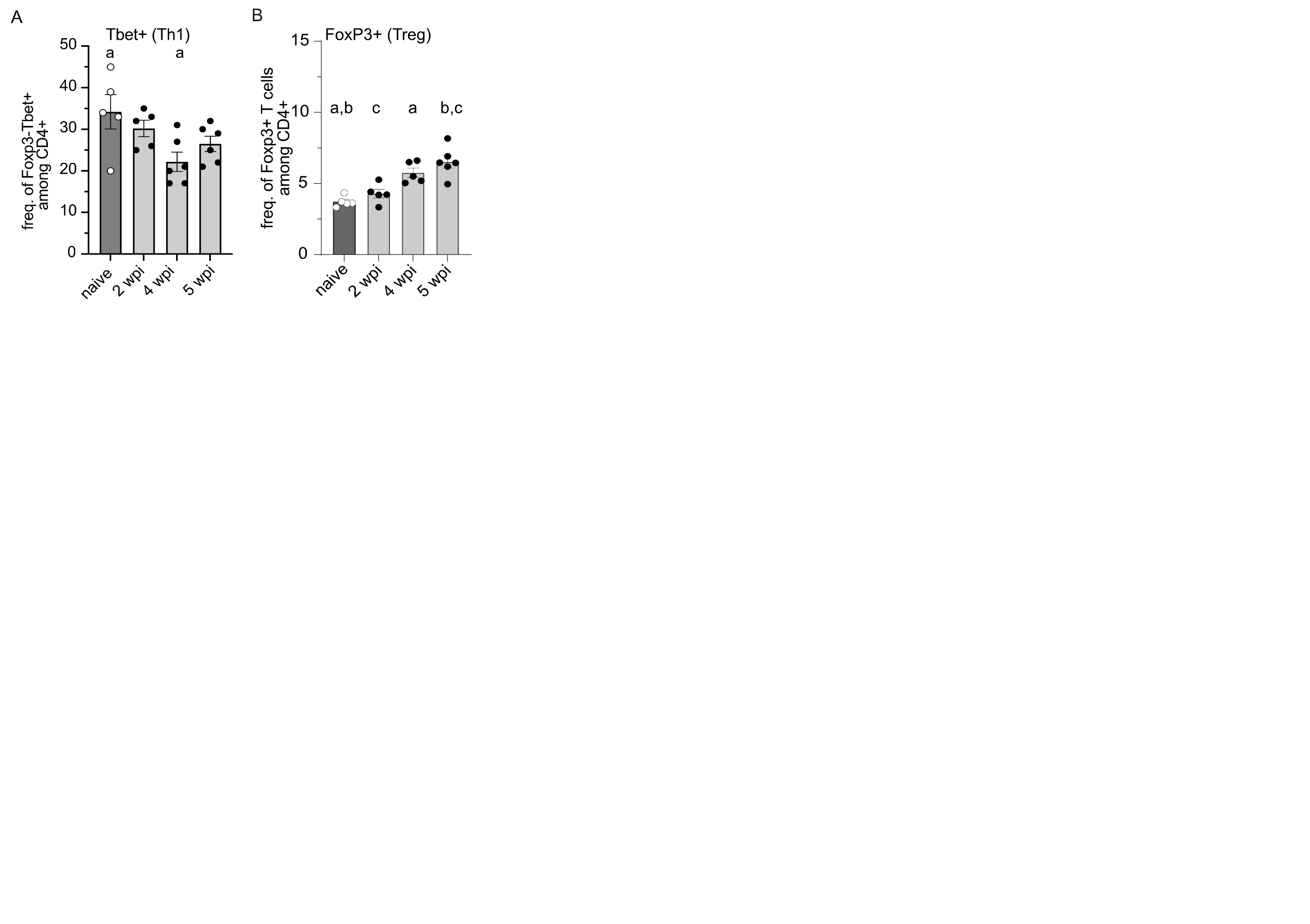


**Suppl. 3.** **Th1 and Tregs in lung tissue following larval A*. suum* migration.** A) Individual Th1 frequencies in lung tissue lymphocytes for n=5-6 animals per group and presented as mean ± SEM. Univariate ANOVA GLM with Bonferroni´s multiple comparison test. Different letters indicate statistically significant differences between groups (naïve *vs.* 4 wpi, a= 0.028). B) Individual FoxP3+ (Treg) cell frequencies in lung tissue at 2, 4, and 5 wpi compared to naïve animals presented as mean ± SEM of n=5-6 animals per group. Univariate ANOVA GLM with Bonferroni´s multiple comparison test. Small letters indicate statistically significant differences between groups (naïve *vs.* 4 wpi, a= 0.005; naïve *vs.* 5wpi, b< 0.001; 2 *vs.* 5 wpi, c=0.003).


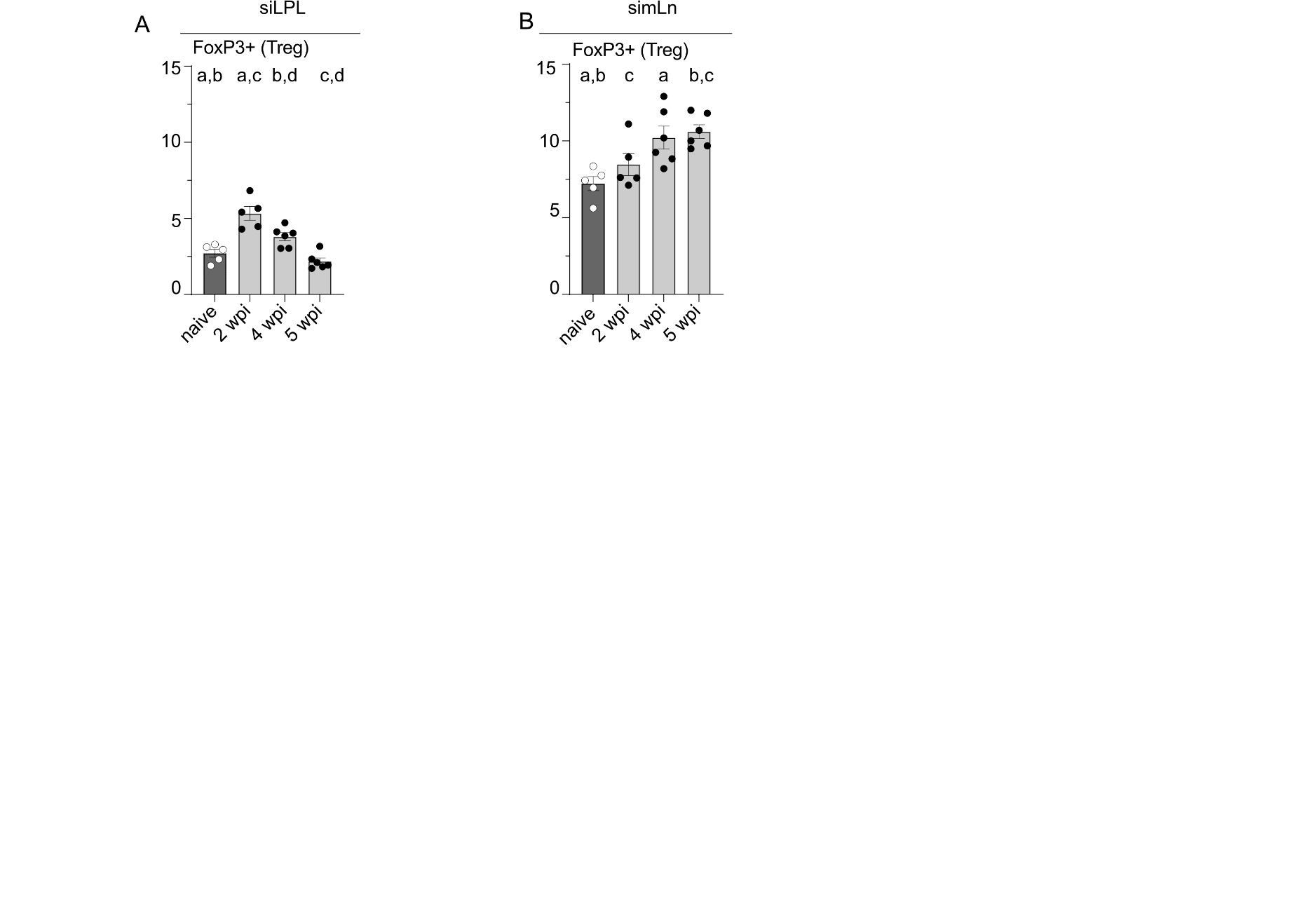


**Suppl. 4.** **Tregs increase in simLn and decrease in siLPL during *A. suum* pre-patency**. A) Individual FoxP3+ (Treg) cell frequencies in small intestinal (jejunal) lamina propria lymphocytes (siLPL) at 2, 4, and 5 wpi compared to naïve animals presented as mean ± SEM of n=5-6 animals per group. Univariate ANOVA GLM with Games-Howell´s multiple comparison test. Small letters indicate statistically significant differences between groups (naïve *vs.* 2 wpi, a= 0.012; naïve *vs.* 4 wpi, b= 0.029; 2 *vs.* 5 wpi, c= 0.006; 4 *vs.* 5 wpi, d< 0.001). **B**) Individual FoxP3+ (Treg) cell frequencies in small intestinal (jejunal) mesenteric lymph nodes (simLN) lymphocytes at 2, 4, and 5 wpi compared to naïve animals presented as mean ± SEM of n=5-6 animals per group. Univariate ANOVA GLM with Bonferroni´s multiple comparison test. Small letters indicate statistically significant differences between groups (naïve *vs.* 4 wpi, a= 0.32; naïve *vs.* 5 wpi, b=0.009; 2 *vs.* 5 wpi, c= 0.038).


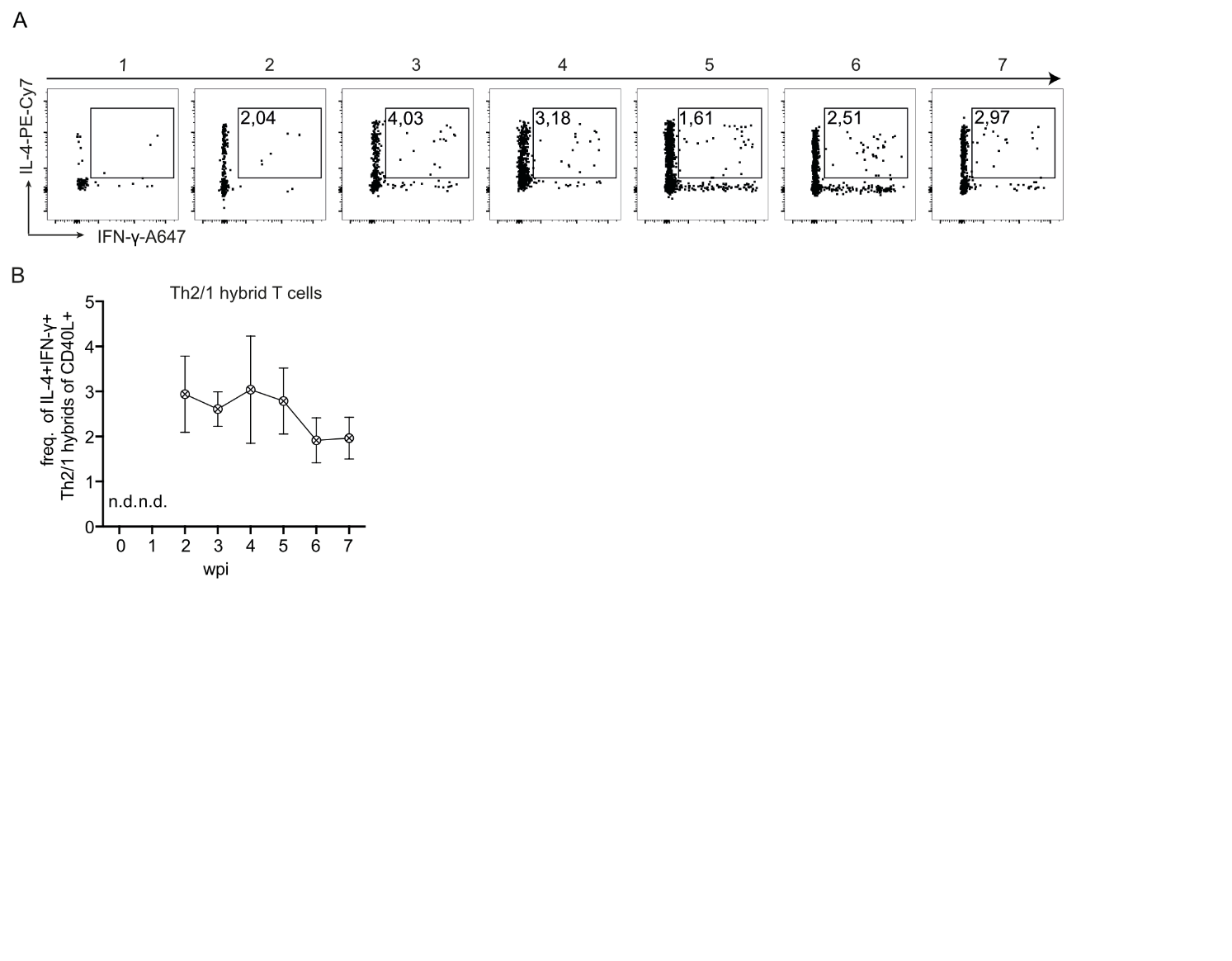


**Suppl. 5. Parasite-specific Th2/1 hybrid cell levels remain constant during primary *A. suum* infection.** A) Exemplary dot plots of CD40L-enriched *A. suum*-specific CD4+ T cells in blood co-expressing IFN-γ and IL-4 0 to 7 wpi and summarized in B) as frequencies of IFN-γ and IL-4 co-producing parasite-specific CD4+ T cells. Samples were controlled for background signals (- cell numbers from unstimulated (w/o) samples) and presented as mean ± SEM from 0-7 wpi. Frequencies not depicted (n.d.) due to very low numbers (<100 events) of parasite-specific CD40L+ T cells in PBMC before (0 wpi) and one week after infection (1 wpi). C) Individual frequencies of IFN-γ-producing (white circle) and IL-4-producing (black circle) parasite-specific CD4+ T cells were controlled for background signals (- cell numbers from unstimulated (w/o) samples) and presented as mean ± SEM at 4 and 5 wpi.
